# Supplementary material for: Mechanical Stress Improves Fat Graft Survival by Promoting Adipose-Derived Stem Cells Proliferation
Source: Int J Mol Sci. 2022 Oct 6;23(19):11839. doi: 10.3390/ijms231911839 (PMC9569524; doi:10.3390/ijms231911839)
Supplement: Supplementary file 1 [file ijms-23-11839-s001.zip › ijms-1932206-supplementary.pdf]

Table S1. Sequences of mouse primer used for qRT-PCR assay.

Gene	Primer sequence (5'-3')	
	Forward	Reverse
Pparg2	TCG CTG ATG CAC TGC CTA TG	GAG AGG TCC ACA GAG CTG ATT
Cebpa	CAA GAA CAG CAA CGA GTA CCG	GTC ACT GGT CAA CTC CAG CAC
Scd1	GCT GGA GTA CGT CTG GAG GAA	TCC CGA AGA GGC AGG TGT AG
Fasn	GGA GGT GGT GAT AGC CGG TAT	TGG GTA ATC CAT AGA GCC CAG
Lpl	GGG AGT TTG GCT CCA GAG TTT	TGT GTC TTC AGG GGT CCT TAG
Fabp4	AAG GTG AAG AGC ATC ATA ACC CT	TCA CGC CTT TCA TAA CAC ATT CC
